# Effects of negative air ions (NAIs) on *Leishmania major*: A novel tool for treatment of zoonotic cutaneous leishmaniasis (ZCL)

**DOI:** 10.1371/journal.pone.0274124

**Published:** 2022-09-08

**Authors:** Alireza Badirzadeh, Mehdi Najm, Andrew Hemphill, Maryam Alipour, Hamid Hasanpour, Leila Masoori, Poorya Karimi

**Affiliations:** 1 Department of Parasitology and Mycology, School of Medicine, Iran University of Medical Sciences, Tehran, Iran; 2 Department of Medical Laboratory Sciences, Faculty of Paramedical Sciences, Lahijan Branch Islamic Azad University, Lahijan, Iran; 3 Department of Infectious Diseases and Pathobiology, Vetsuisse Faculty, Institute of Parasitology, University of Bern, Bern, Switzerland; 4 Department of Parasitology and Mycology, School of Allied Medical Sciences, Ilam University of Medical Sciences, Ilam, Iran; 5 Razi Herbal Medicines Research Center, Lorestan University of Medical Sciences, Khorramabad, Iran; INRS, CANADA

## Abstract

**Background:**

Cutaneous leishmaniasis (CL) is a Neglected Tropical Disease (NTD) that causes high morbidity in the tropics and sub-tropics. Despite the remarkable advancements in the treatment of CL, the available therapeutics are far from ideal and also cause serious adverse side effects. Negative air ions (NAIs) generators are widely available for domestic and industrial uses. Several studies have reported on positive effects of NAIs therapy on human health as a non-pharmaceutical treatment for respiratory disease, allergy, or stress-related health conditions, including infectious diseases. To our knowledge, no studies have examined the effectiveness of the NAIs therapy against *Leishmania* parasites. The aims of this study were to investigate the effect of NAIs therapy on *Leishmania major* (*L*. *major*) the causative agent of CL in *in vitro* and in a murine model.

**Methodology/Principal findings:**

*In vitro* anti-leishmanial effects of NAIs therapy were measured by parasitological methods. NAIs therapy was assessed *in vivo* in *L*. *major* infected BALB/c mice by measuring the footpad (FP) lesion size and parasite load using metric caliper tool and qPCR, respectively. Immune responses in treated and non-treated mice were assessed by measuring the levels of IFN-γ, IL-4, NO and arginase activity. *In vitro* NAIs therapy significantly decreased the viability of *Leishmania* promastigotes and of amastigotes cultured in macrophages, but did not affect the host cells. NAIs therapy of *L*. *major* infected BALB/c mice resulted in reduced FP lesion size, diminished parasite burden, and importantly decreased induction of IL-4 and arginase activity in the presence of NAIs. In contrast IFN-γ and NO levels were significantly enhanced. NAIs therapy significantly diminished the progression of disease compared to the control group, but was less effective than amphotericin B treatment.

**Conclusions:**

Our study shows that NAIs treatment was effective *in vitro* and in *Leishmania*-infected mice, elicited a T-helper 1 (Th1) response and increased efficient cellular immunity, resulting in a diminished parasite load. Therefore, NAIs therapy can be considered as a useful and safe tool that can contribute to clearing *L*. *major* infections without inducing toxicity in host cells. The applications and mechanisms of NAIs therapy warrant further investigation especially in humans suffering from CL.

## Introduction

Cutaneous leishmaniasis (CL) is one of the major neglected tropical diseases (NTDs) endemic in the different countries of tropics and subtropics region of the globe, especially in poverty-stricken countries with an estimated worldwide incidence of approximately 1.5 million cases annually [1–3]. By far, CL is the most prevalent form of leishmaniasis, with various symptoms ranging from one single self-healing lesion to acute or chronic metastatic infection. This vector-borne disease is caused by several *Leishmania* (*L*) species, which are intracellular protozoan parasites [4, 5]. In the Old World, the main etiologic causative agents of CL are *Leishmania major*, *L*. *tropica*, *L*. *aethiopica*, and recently *L*. *infantum* [2, 6, 7].

To date, control of CL in humans largely depends on chemotherapy. However, various disadvantages restrict the use of the currently available drugs [2]. Compounds for the treatment of leishmaniasis are pentavalent antimonials, amphotericin B, pentamidine and miltefosine. Adverse effects include musculoskeletal pain, gastrointestinal disturbances, and mild to moderate headache, dyspnea, erythema, cardiotoxicity and nephrotoxicity. Besides, resistance formation has resulted in treatment failures [2, 8, 9]. Thus, novel ways of CL treatments are warranted [8, 10, 11].

Air in the atmosphere is a mixture of various gases (oxygen, carbon dioxide, nitrogen, H_2_O vapor, and trace amounts of other gases) which can be easily ionized. When these gas molecules and/or atoms release or acquire an electron, they energize and become charged and are called air ions. These are commonly divided into negative air ions (NAIs) and positive air ions (PAIs) [12–14]. NAIs have acquired an electron, while PAIs have release an electron [12–14]. Air ions are generated through natural phenomena such as alterations in the atmosphere and/or weather. They are also produced industrially and commercially through air ionizers, which are widely available for home or industrial uses. Several studies have shown that air ions, and especially NAIs, have various positive biological effects and actions [12]. They have been applied for more than a century for cleaning the air of various aerosol particles, for killing of distinct airborne microbes, and elimination of different odors [12, 15–17]. Exposure to NAIs has been experimentally linked to declining the viability of various microbial cells, and NAIs also exhibit lethal effects on several microorganisms including gram-positive and gram-negative bacteria such as *Staphylococcus aureus*, *Vibrio cholerae*, *Salmonella* sp., *Escherichia coli*, and also affected the fungal pathogen *Candida albicans* and the non-parasitic worm *Caenorhabditis elegans* [15, 18]. However, the effects of NAIs on distinct infectious parasites are unknown. The mechanism action of NAIs against microorganisms has been related to cell agglutination, to physical displacement of cells, or to ion discharge [18]. NAIs react with oxygen, resulting in the production of highly reactive oxygen radicals that exhibit bactericidal effects [18, 19].

To date there is only very limited documented data on the sensitivity of microorganisms to NAIs, and no studies have assessed the potential anti-parasitic effects of NAIs. Thus, we present the first report on anti-parasitic characteristics of NAIs therapy, by demonstrating the efficacy against *L*. *major in vitro* and in a BALB/c infection model.

## Material and methods

### Parasite culture

*Leishmania major* (MRHO/IR/75/ER) promastigotes were kindly provided by Prof. Sima Rafati (Department of Immunotherapy and *Leishmania* Vaccine Research, Pasteur Institute of Iran, Tehran, Iran). *L*. *major* promastigotes were cultured at room temperature (∼26°C) in RPMI-1640 medium (Sigma- Aldrich Chemicals; Germany) supplemented with 10% heat-inactivated fetal calf serum (FCS) and 100 μg/mL penicillin/ streptomycin, pH 7.4. They were sub-cultured daily. In vivo studies were carried out by inoculation of 2×10^6^ metacyclic promastigotes into the footpad (FP) of BALB/c mice, and after four weeks parasites were isolated from lymph nodes (LNs) of infected mice. The isolated LNs from BALB/c mice were homogenized and were cultured as described above. Parasites were kept in a virulent state by five successive passages in BALB/c mice.

### Macrophage cell-line culture

The murine macrophage cell line J774A.1 (ATCCTIB-67TM) was obtained from the Pasteur Institute of Iran, Tehran, Iran. J774A.1 was cultured in RPMI 1640 medium with 10% FCS and 100μg/mL penicillinand/streptomycin at 37°C and 5% CO_2_ (Memert; USA), and passages were carried out every two or three days. After thawing frozen J774A.1 cells, were passaged three times for getting sufficient cells. In general, we used passage number six for all *in vitro* experiments.

### Negative air ions (NAIs)

To investigate the effects of NAIs upon *L*. *major*, the NAIs generator (Neotec, XJ-2100) was applied. Prior to applying the NAIs generator, the UV lamp was covered with thick aluminum foil to avoid UV exposure. The NAIs generator was placed at a height of one meter above the floor, and its independently controlled fan was turned on for increasing the air circulation. The experiment was done in closed sterile laminar hood (HO04-M, Pars Azma Co., Tehran, Iran) and the system ventilation was turned off during the experiment. The concentration of NAIs was constantly kept at 10^6^ NAI/cm^3^. For recording the ion concentration in the air box, a portable ion counter (Air Ion Counter IC 1000, Ion Trading, Tokyo, Japan) was applied [17, 20].

### *In vitro* anti-promastigote effects of NAIs

For evaluation of anti-promastigote effects of NAIs, *L*. *major* promastigotes (1×10^6^/well) were seeded into a 96-well flat-bottom micro-titer plate in the presence of a constant current of 10^6^ NAI/cm^3^, and were maintained for 2, 4, 6 and 16 h at 24–26°C. Promastigotes maintained in the absence of NAIs were used as negative control. Parasite viability was determined by the Trypan blue dye (Sigma–Aldrich, St. Louis, USA) exclusion method [21]. Briefly, 0.1 mL of *Leishmania* promastigotes from all experimental groups were added to 0.1 mL Trypan blue, and both living (non-stained) and dead (stained) cells were counted in a Neubauer chamber. Experiments were performed in triplicate wells and after 2, 4, 6 and 16 h NAIs treatment, viable *Leishmania* promastigote counts were determined. Amphotericin B (Sigma‐Aldrich, St. Louis, USA) (1.9 μg/mL) and PBS 1X were applied as positive (reference drug) and negative controls, respectively for 2, 4, 6 and 16 h at 24–26°C. As results for these controls were identical at all timepoints, only the 16 h timepoint is shown.

### Assessment of J774A.1 macrophage viability

Viability evaluation of the NAIs on J774A.1 macrophages was performed as for *L*. *major* promastigotes but in atmospheric CO_2_ condition (37°C and 5% CO_2_). Briefly, an aliquot of 0.1 mL of macrophages from all experimental groups were added to 0.1 mL Trypan blue. After mixing, both non-stained and stained cells were counted in a Neubauer chamber. Experiments were performed in triplicate wells and after 2, 4, 6 and 16 h NAIs treatment, viable J774A.1 macrophage counts were determined. Amphotericin B (Sigma‐Aldrich, St. Louis, USA) (1.9 μg/mL) and PBS 1X were applied as positive (reference drug) and negative controls, respectively for 2, 4, 6 and 16 h. As results for these controls were identical at all timepoints, only the 16 h timepoint is shown.

### Assessment of intracellular parasite viability

J774A.1 macrophages (5×10^4^ cells) were seeded into 96-well flat-bottom micro-titer plates and incubated for 24 h at 37°C in 5% CO_2_. *L*. *major* promastigotes were used to infect the macrophages with parasite-to-host cell ratio of 1:10 followed by culture for 24 h. Free and dead parasites were removed by washing three times in serum-free RPMI-1640 medium. Finally, infected macrophages were exposed to NAIs with constant current of 10^6^ NAI/cm^3^ during 2, 4, 6 and 16 h in atmospheric CO_2_ condition (37°C and 5% CO_2_). Parasite viability was determined by the Trypan blue dye (Sigma–Aldrich, St. Louis, USA) exclusion method [21]. Briefly, 0.1 mL of *cells* from all experimental groups were added to 0.1 mL Trypan blue, and both living (non-stained) and dead (stained) cells were counted in a Neubauer chamber. The reference drug amphotericin B (1.9 μg/mL) and PBS 1X were applied as positive and negative controls, respectively for 2, 4, 6 and 16 h under the same conditions. As results for these controls were identical at all timepoints, only the 16 h timepoint is shown.

### Assessment of *Leishmania* parasite intracellular viability

In order to investigate whether NAIs treatments effectively killed intracellular amastigotes, the infected J774A.1 macrophages (5×10^4^ cells) from the previous step were washed thrice with fresh Schneider’s complete medium. Then, 25 μL of Schneider’s medium containing SDS 0.05% was added to each well for lysis of the *Leishmania* infected macrophages. After shaking the plate, cultures were washed and the culture medium with lysis buffer was replaced with Schneider’s Drosophila medium containing 10% FCS. Next, the plates were incubated at 23–25°C for five days to allow intracellular amastigotes to differentiate into motile promastigotes. Finally, by using a Neubauer chamber promastigote numbers were determined [22–24].

### Ethics statement

The current research has received ethical approval from National Institute for Medical Research Development (NIMAD) in Iran (ethical code: IR.NIMAD.REC.1398.285). Procedures with animals in this experiment was done according to the guidelines of the Specific National Ethics for Biochemical Research issued by the Research and Technology Deputy of the Ministry of Health and Medical Education (MOHME) of Iran (issued 2005).

### Mice

*In vivo* experiments in BALB/c mice were performed in a way to minimize suffering. Seventy five female BALB/c mice (6 weeks old; weigh 18 to 22 g) were purchased from the Animal Laboratory of the Pasteur Institute of Iran, Tehran, Iran. Animals were housed in clean ventilated-plastic cages in a controlled animal care facility at a temperature of 22±2°C, humidity 55%, and 12 h of light-dark cycles. Mice had free access to food and water. Infection with *L*. *major* was done by subcutaneous (s.c) injection of 2×10^6^ metacyclic promastigotes from the stationary growth phase in the right hind FP.

### Safety assessment of NAIs treatments in BALB/c mice

Potential toxic effects of NAIs, and potential adverse effects due to manipulation of BALB/c mice were assessed in five experimental groups (G_1_-G_5_). G_1_, G_2_, G_3_ and G_4_ were exposed to 10^6^ NAI/cm^3^ during 2, 4, 6 and 16 h, respectively, and G5 remained unexposed. For the treatments cages were placed in a closed sterile laminar hood (HO04-M, Pars Azma Co., Tehran, Iran) and the system ventilation was turned off during the experiment. Animals were kept at a temperature of 22±2°C, humidity 55%, and 12 h of light-dark cycles. Mice had free access to food and water through the NAIs therapy. All animals were monitored carefully for seven days and vital signs including body weight, sound sensitivity, diarrhea, mental consciousness such as sleepiness, and shedding of body hair were recorded during and up to ten days after NAIs therapy.

### NAIs therapy in *L*. *major*-infected BALB/c mice

Potential protective effects of NAIs therapy in *L*. *major* infected BALB/ mice were assessed in seven experimental groups (G_1_-G_7_) of ten mice each. Infection was done by s.c injection of 2×10^6^ metacyclic promastigotes from the stationary growth phase in the right hind FP. NAIs therapy was started on day 21 post-infection (p.i), when CL lesion development was obvious. G_1_-G_4_ were exposed to a treatment that included exposure to NAIs for 2, 4, 6 and 16 h per day, respectively, 5 times per week during 4 weeks. G_5_ remained untreated (negative control). G_6_ was treated with amphotericin B (8 mg/kg) injected by intraperitoneal (IP) route twice daily during 28 days, and G_7_ received PBS injected by the intralesional (IL) route. As indicated above, the cages were placed in a closed sterile laminar hood and the system ventilation was turned off during the experiment. Animals were kept at a temperature of 22±2°C, humidity 55%, and 12 h of light-dark cycles. Mice had free access to food and water through the NAIs therapy.

FP lesion sizes in the injection site were monitored twice weekly and variations in lesion size were recorded once per week using metric caliper until day 50 p.i. After the end of treatment, five mice from each group were randomly selected and sacrificed by cervical dislocation. The infected FPs, inguinal LNs and spleens were isolated and evaluated for parasite load, IL-4-, IFN-γ- and NO-levels, and arginase enzymatic activity.

### Quantification of *Leishmania* parasite load

The parasite load was measured by two methods: quantitative real-time PCR (qPCR) and limiting-dilution assay as described previously [2, 25, 26]. For qPCR, infected FPs and inguinal draining LNs were aseptically isolated and homogenized. Genomic DNA was extracted via phenol-chloroform-isoamyl alcohol (PCI) [27], and the DNA concentration was determined using a Nano drop (ND-1000, USA) spectrophotometer at 260 nm. 40 ng of extracted genomic DNA was used for qPCR. Two different sets of primers were used, which targeted a specific region of the kinetoplastid minicircle DNA (kDNA) of *L*. *major* including RV1 and RV2 primers (Forward: 5′-CTTTTCTGGTCCCGCGGGTAGG-3′ and Reverse: 5′-CCACCTGGCCTATTTTACACCA-3′). Total genomic *L*. *major* DNA from 3×10^7^ parasites was serially diluted in seven-fold steps in order to draw the standard curve. All qPCR reactions were done in duplicate. For quantification of parasite load by limiting dilution assay, five mice from each group were sacrificed separately. After aseptical isolation of infected FPs and inguinal LNs, they were homogenized in RPMI-1640 medium. The homogenates were serially diluted (22 serial dilutions) in RPMI-1640 medium with 10% fetal bovine serum (FBS) in 96-well microtitration plates in triplicate, and the plates were incubated at 26°C for 2 weeks. During two weeks in the culture media the presence of motile promastigotes was recorded and parasites were counted microscopically. The *Leishmania* parasite load was calculated as following formula [25, 28]: Parasite load = (-log parasite dilution/Organ’s weight)

### Measurements of IL-4 and IFN-γ in splenocyte culture supernatants

IL-4 and IFN-γ expression was assessed in splenocyte culture supernatants as previously described [2, 29]. 50 days p.i. (after the end of NAIs therapy), five mice from each group were sacrificed and their spleens were isolated and homogenized in DMEM phenol red-free medium (Sigma- Aldrich Chemicals; Germany) supplemented with 10% heat inactivated FBS. After three days for IL-4 and five days for IFN-γ, the cytokines productions in response to concanavalin A (Con A, 5 μg/mL) and medium alone (no antigen), in the supernatants were assessed using commercial ELISA kits (R&D, Minneapolis, MN, USA), as described previously [2, 29].

### Arginase activity and NO measurements

Arginase activity were carried out in the FPs of those mice used for splenocyte culture preparation. The arginase activity test quantifies the conversion of L-arginine to L-ornithine, as described elsewhere [2, 30]. NO production measurement was carried out by applying the protocol of the Griess Reagent System (Promega, Madison, WI). 100 μl of Griess Reagent [0.1 N (1-naphthyl) ethylenediamine dihydrochloride, 1% sulfanilamide in 5% H_3_PO_4_] was directly mixed with 100 μl of culture supernatant of stimulated splenocytes. After a10 min incubation at room temperature, the colored (azo dye) complex absorbance was measured at an OD = 570 nm. The NO absorbance values of each sample were quantified in terms of standard curve of nitrate [2, 30, 31].

### Statistical analysis

All statistical analyses were performed using Prism 8.0 software (version 8.0; GraphPad Software, Inc 2018, San Diego, CA, USA). One-way ANOVA and Student’s t-test were used for performing the comparison between groups. The association between the cytokines (IFN- γ /IL-4) induction and differences in *Leishmania* parasite load were calculated by applying Spearman correlation method. *P* values <0.05 were considered as statistically significant. The results are shown as the mean ± SD of three consecutive tests with identical results that were carried out in triplicate.

## Results

### Exposure to NAIs impairs the viability of *L*. *major* promastigotes and amastigotes *in vitro*

**Fig 1:**
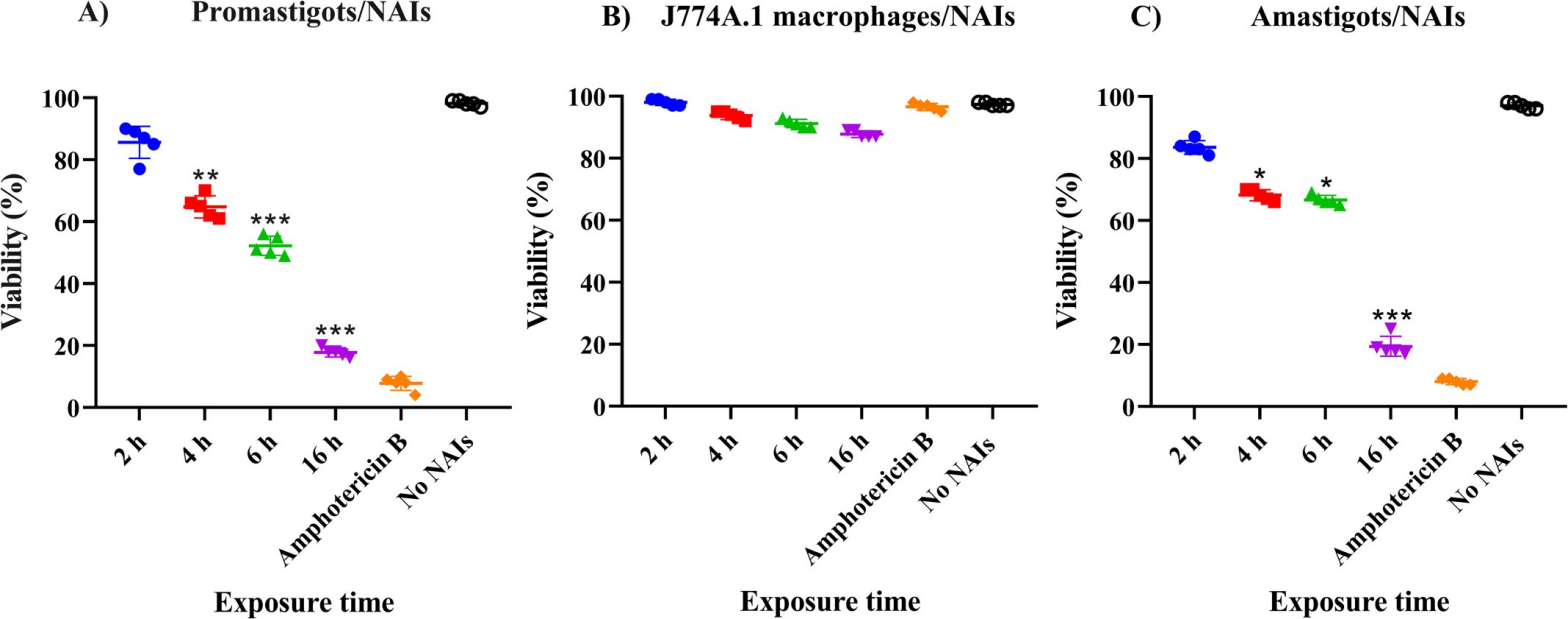
Cell viability and *in vitro* anti-leishmanial effects of NAIs (10^6^ NAI/cm^3^) on *L*. *major* promastigotes (A), the macrophage cell line J774A.1 (B) and on macrophages infected with *L*. *major* amastigotes (C), at exposure times of 2 h, 4 h, 6 h and 16 h. Amphotericin B (1.9 μg/mL) and PBS 1X were applied as positive (reference drug) and negative controls, respectively for 2, 4, 6 and 16 h. As there was no difference in all four time-points, only the results for 16 h exposure are shown. The data are shown as the mean ± SD of three consecutive repeated tests with identical results that were carried out in triplicate, which statistical test was used P value descriptions as in the other figures. (*P < 0.05, **P < 0.01, ***P < 0.001).

The viability of *L*. *major* promastigote forms after exposure to 10^6^ NAI/cm^3^ was determined and expressed in %-age to the non-treated controls (Fig 1A). NAIs reduced *Leishmania* promastigotes viability in a time-dependent manner. While after 2 h, viability of promastigotes was 83%, viability progressively decreased to 10% after 16 h of exposure ((*P*<0.001).

The effects of NAI exposure on the macrophage cell line J774A.1 (Fig 1B) and on *L*. *major* amastigote-infected macrophages (Fig 1C) were also investigated. The viability of intracellular amastigotes inside parasite infected cells was impaired in a time-dependent manner, with the 16 h exposure being the most effective (*P*<0.001), while no loss of viability was seen in non-infected J774A.1 macrophages (P<0.001).

### NAI exposure leads to reduced CL lesion size in BALB/c mice

**Fig 2:**
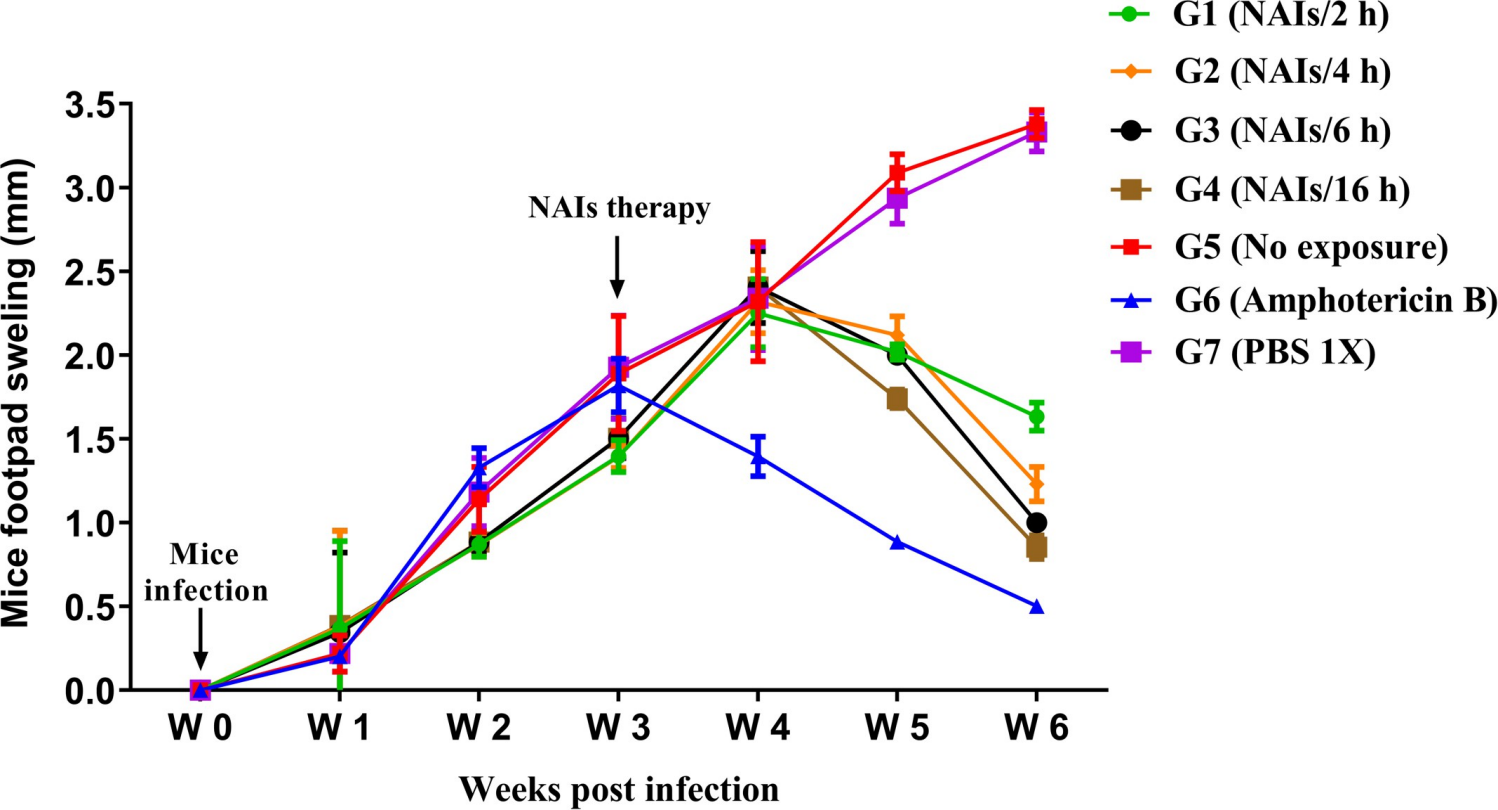
Experimental CL lesion development or swelling of right footpad (FP) in *Leishmania major* (2×10^6^ metacyclic promastigotes) infected BALB/c mice before and after NAIs therapy (after the end of treatment, five mice from each group were randomly selected). The CL lesion size was reported using a metric caliper tool by measuring the increase in right infected FP thickness/width from 1 to 6 weeks p.i. The results of one out of three independent experiments are shown, all presenting similar outcomes. Results are elucidated as the mean ± SE and student t-test was used for statistical analysis (*P < 0.05, **P < 0.01, ***P < 0.001).

To evaluate the effect of NAIs therapy on the CL lesion in experimentally infected BALB/c mice, animals were infected subcutaneously by inoculation of *L*. *major* promastigotes into the FP. Following infection CL lesion development and swelling were monitored weekly for about 6 weeks (Fig 2). NAIs therapy was initiated three weeks p.i. All experimental groups undergoing NAIs therapy (G1-G4) as well as the amphotericin B treated control group (G6) exhibited smaller lesion sizes in comparison to both negative controls groups (PBS treatment or no treatment) (Fig 2). Note that G4 (NAIs/ 16 h) among the test groups and G6 (Amphotericin B) as the positive control among all of the groups had the smallest FP CL lesion size (*P*<0.001).

### NAI therapy leads to reduced *Leishmania* load in the LNs and FP of infected mice

**Fig 3:**
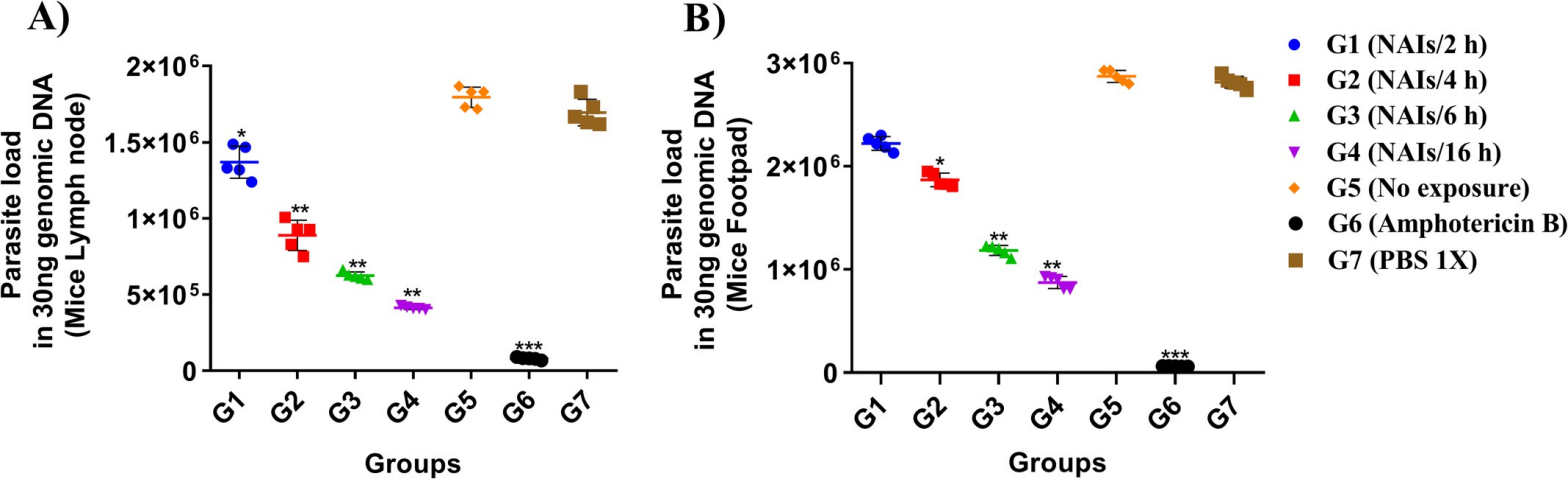
*Leishmania major* parasite loads in the draining inguinal lymph nodes (LNs) (A) and footpad (FP) (B) of BALB/c mice after NAIs therapy at six weeks post CL infection by using qPCR (after the end of treatment, five mice from each group were randomly selected). All data are reported as the mean ± SD, five mice per group. One of three independent experiments is shown, all presenting similar outcomes (*P < 0.05, **P < 0.01, ***P < 0.001).

**Fig 4:**
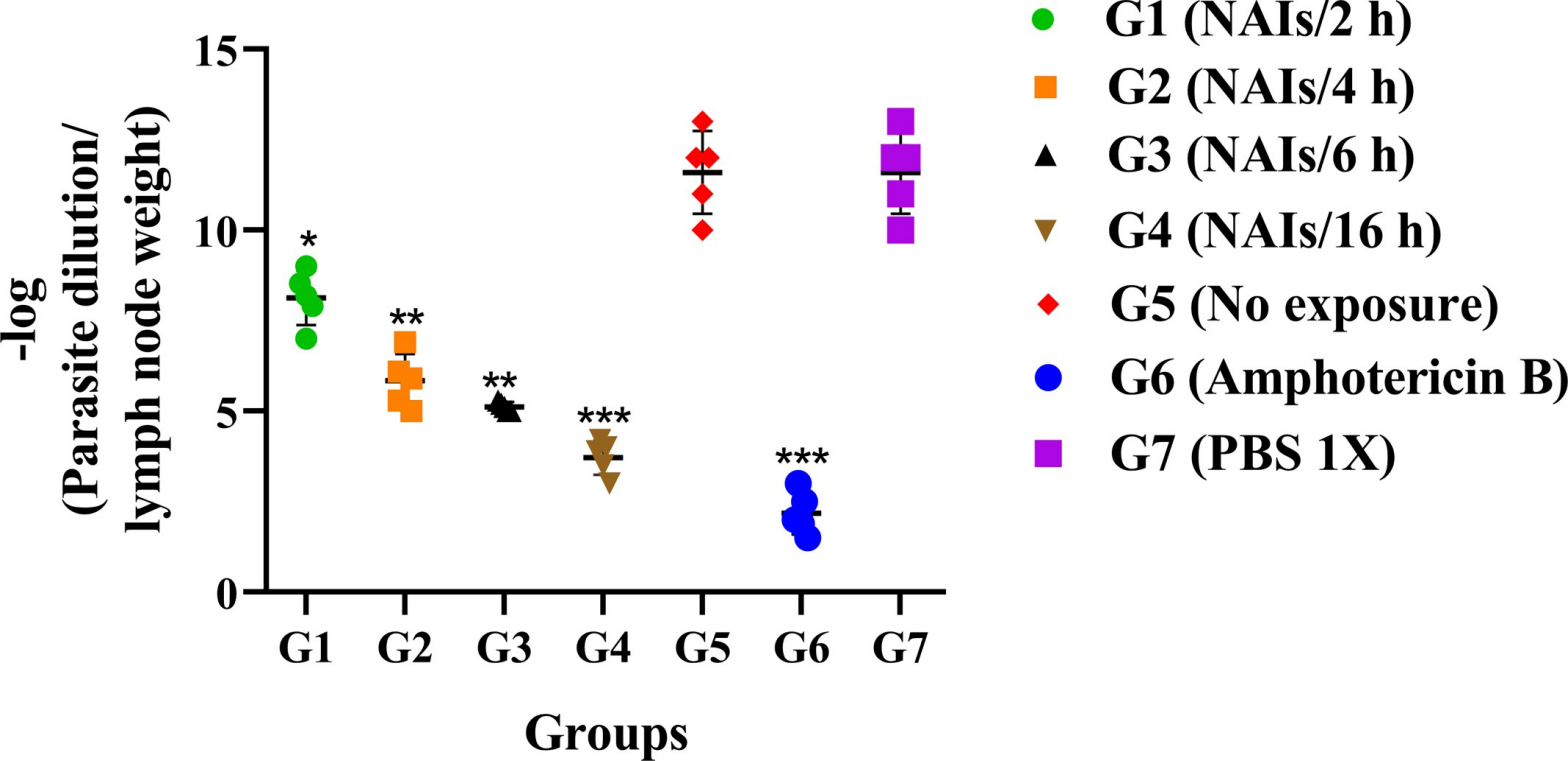
*Leishmania major* parasite loads in the draining inguinal lymph nodes (LNs) of different mice groups following NAIs therapy at six weeks post CL infection by using conventional limiting dilution assay (microtitration) (after the end of treatment, five mice from each group were randomly selected). All data are reported as the mean ± SD, five mice per group. Data of one out of three independent experiments is shown, all three presenting similar outcomes (*P < 0.05, **P < 0.01, ***P < 0.001).

The number of viable *Leishmania* parasites in the LNs and FP of infected BALB/c mice was assessed using quantitative real-time PCR (qPCR) (Fig 3) and conventional limiting dilution assay (microtitration) (Fig 4). Assessments were done at six weeks p.i. for all groups. As shown in Figs 3 and 4, the *L*. *major* parasite load was remarkably reduced and positively correlated to the duration of treatments (G1 to G4) compared to negative control groups G5 (no exposure). Groups G4 (16 h) and G6 (Amphotericin B as positive reference drug), had the lowest parasite loads in comparison with other groups (*P*<0.001). No differences were seen in the number of viable *Leishmania* parasites in the LN and FP of the infected BALB/c mice (Figs 3 and 4).

### NAIs therapy of infected BALB/c mice affects cytokine responses

**Fig 5:**
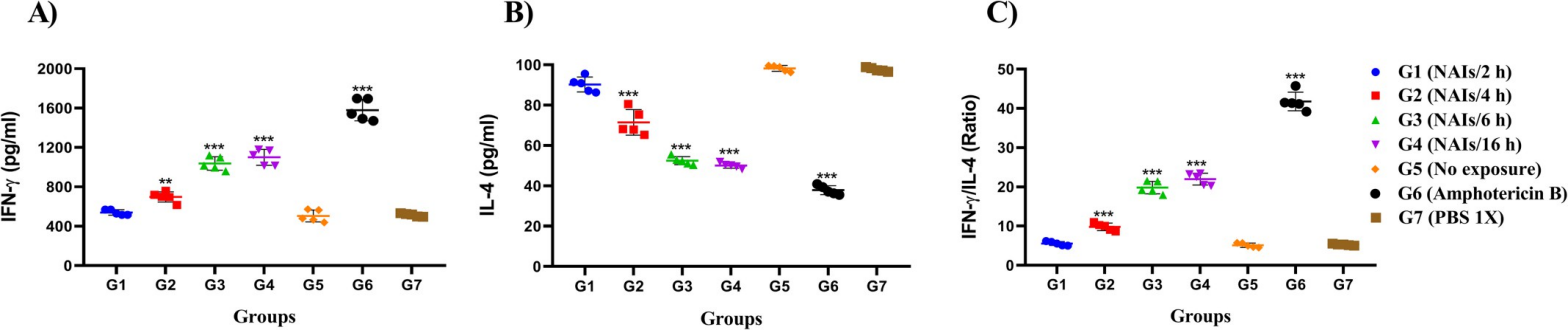
Evaluation of IFN- ɣ (A) and IL-4 (B) production in the supernatant of stimulated splenocytes of *L*. *major*-infected BALB/c mice in NAIs therapy groups (G1-G4) and controls (G5, G6 and G7), and IFN- ɣ/IL-4 ratio (C) (after the end of treatment, five mice from each group were randomly selected). All data are presented as the mean ± SD, five mice per group. The data show the results of one out of three independent experiments presenting similar outcomes (*P < 0.05, **P < 0.01, ***P < 0.001).

IFN- ɣ and IL-4 production by stimulated splenocytes from all experimental groups at 6 weeks p.i. were measured (Fig 5). As shown in Fig 5, splenocytes from mice treated with amphotericin B (G6) and three test groups G2, G3 and G4) exhibited higher levels of IFN-ɣ in medium supernatants compared to splenocytes from the negative control group *(P*<0.001). Conversely, significantly lower levels of IL-4 were measured in culture supernatants of splenocytes of the amphotericin B group (G6) and all groups receiving NAI therapy compared to the non-treated control groups (G5 and G7). Increase of IFN- ɣ and decrease of IL-4 levels occurred in a time-dependent manner (Fig 5A and 5B). The IFN- ɣ/IL-4 ratio (Fig 5C) was significantly higher in the amphotericin B treated group (G6), and in groups G2, G3, and G4, respectively compared to the negative controls (G5 and G7).

### Effects of NAIs therapy on arginase activity and NO production in *L*. *major* infected BALB/c mice

**Fig 6:**
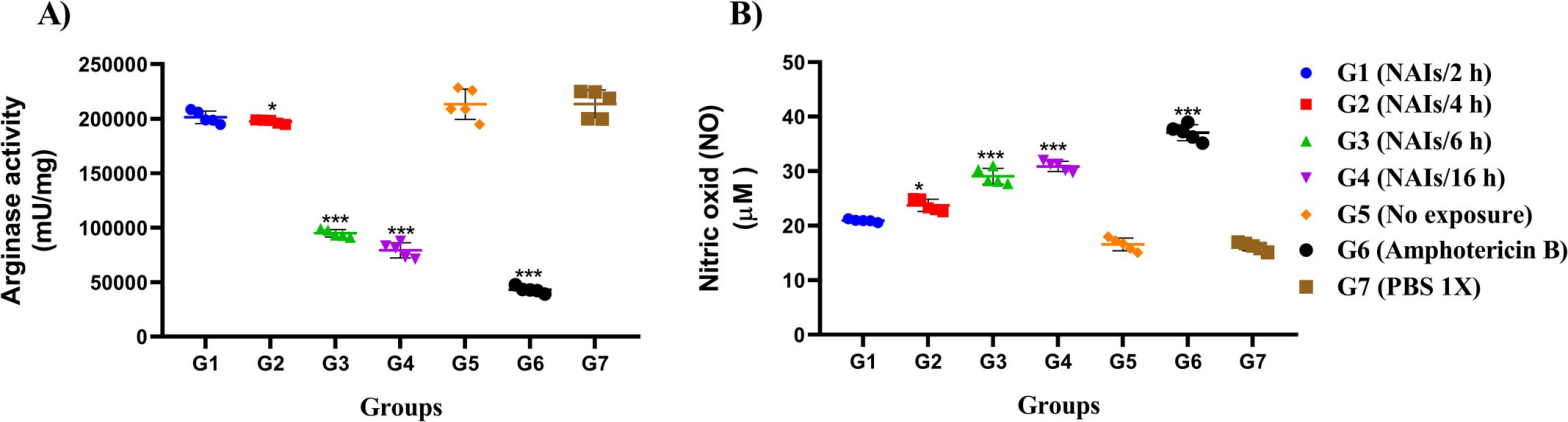
Arginase activity and nitric oxide (NO) production in *L*. *major* infected BALB/c mice undergoing NAIs therapy. Arginase activity (mU/mg) (A) and NO production (μM) (B) were assessed by using microplate method and Griess assay, respectively (after the end of treatment, five mice from each group were randomly selected). All data are shown as the mean ± SD, five mice per group. The data shown are from one representative out of three independent experiments with similar outcomes (*P < 0.05, **P < 0.01, ***P < 0.001).

Killing or long-term survival of intracellular *Leishmania* parasites inside host cells are largely mediated by inducible nitric oxide synthase (iNOS) and arginase activities. In mice treated with NAIs therapy during 6 and 16 h per day (G3, G4), arginase activity was significantly decreased, while no effects were evident in the 2 and 4 h per day treatment groups (Fig 6A). Conversely, significantly increased NO levels were measured in G2, G3 and G4, and NO levels increased upon prolonged treatment duration (Fig 6B).

## Discussion

Although various studies have shown that NAIs can enhance air quality and have beneficial effects on human and animal health, the effects of NAIs on intracellular parasites have not been investigated so far [15]. We here report, for the first time, that NAIs exposure affects extracellular *Leishmania* promastigotes and also intracellular amastigotes, that NAIs therapy has an impact on key immune parameters in *L*. *major* infected BALB/c mice, and leads to reduced CL lesion size.

NAIs have been previously demonstrated to have a lethal effect on several other microorganisms [32] especially bacteria and fungi [18, 32]. For instance *Candida albicans*, one of the major medically important fungi, was shown to be sensitive to NAIs treatment [18]. A study on the effects of electrically generated NAIs on the nematode *C*. *elegans* demonstrated that the treatment reduced the development period of the worm, diminished its lifespan, enhanced apoptosis and shortened the worm brood size. [15]. However, the effects of NAIs treatment on *Leishmania*, and how promastigotes and intracellular amastigotes are affected by NAIs treatment *in vitro*, is not known. The action of the electric field that is generated through NAIs exposure might play an important role [33]. Several experiments suggested that NAIs are primarily composed of free electrons which react directly with oxygen to produce free radicals, yielding toxic effects [18, 19]. The NAIs activity against bacteria has been attributed to the physical displacement of bacteria, cellular agglutination, and to an effect associated with the ion discharge itself. The ion discharge gives rise to oxygen radicals. These oxygen radicals interact with oxygen (O_2_) and carbon dioxide (CO_2_) to generate distinct anions such as O_3_^-^, O_2_^-^and CO_3_^-^ [34, 35]. The bactericidal effects of NAIs have been related to the O_2_^-^and CO_3_^-^ ions. Importantly, the generation of ozone, nitric oxide and nitrous oxide [34] is also possible. In *Pseudomonas aeruginosa* the main principal cause of NAIs-induced bacterial inactivation was exposure to ozone but in *Mycobacterium parafortuitum* electroporation ending from exposure to the electric field was postulated to be the major principal cause of cell death [18, 33, 36]. Similar mechanisms might contribute to the killing of *Leishmania* promastigotes as well as intracellular amastigotes.

NAIs therapy in BALB/c mice infected in the FP with *L*. *major* resulted in diminished CL lesion size and in reduced parasite load in the FP and LNs of treated versus non-treated mice. The treatment was most efficient when applied for 6 and 16 h per day (G3 and G4, respectively). In addition, the continuous NAIDs treatment applied in this study was safe, as no detrimental effects were noted in any of the animals exposed to treatment. *In vivo* efficacy of any treatment is often associated with immunity, and we demonstrate an increased production of IFN-γ and iNOS in NAIs-treated mouse splenocytes, which indicates that a T-helper-1 (TH1)-biased immune response is induced. Conversely, production of IL-4 and arginase, two indicators of T-helper-2 (TH2) activation were decreased. Under normal circumstances, *Leishmania* parasites residing within infected murine macrophages inhibit the induction of TH1-inducing cytokines such as IFN-γ and cell mediated responses and NO production, but modulate the host cell to produce IL-4 and arginase, in order to promote parasite survival. One of the important points in the current study relates to the effects of NAIs therapy on parasite reduction. According to our data, NAIs therapy can reduce parasite replication and spread. Thus, the effects on the TH1 responses can be due to reduction in the parasite suppression, rather than TH1-inducing cytokines induction.

Other effects of NAIs therapy on immune cells have been described. For instance, NAIs therapy applied after methylcholan-threne carcinogenesis in mice increased the activity of natural killer cell (NK) cell activity and led to tumor regression and prolonged survival [19, 37]. NAIs were demonstrated to increase the negative charges of the NK (natural killer) cell membrane and associated membrane proteins, thereby leading to NK cell activation [19]. NK cells are known to act as the first line of defense in the immune reaction after *Leishmania* infection. They are potent producers of IFN-γ, and depletion of mouse NK) cells dramatically enhances susceptibility against infection of normally resistant mice [38]. *In vitro* studies have shown that promastigotes are directly lysed by NK cells, but NK cell activity is also modulated by promastigotes. In human patients suffering from acute cutaneous leishmaniasis, impaired NK responses and reduced NK cell numbers have been observed, and studies in other animal models have shown that NK cells play a key role in the induction and direction of the immune response. Thus, the beneficial effects of NAI therapy observed herein could also be based on effects on NK cells [38].

Although this study demonstrated that NAI therapy reduced *L*. *major* infection in BALB/c mice and delayed CL lesion development, NAI therapy did not lead to the complete clearance and destruction of the parasite in the lesion site. According to studies in humans, ulcerative CL lesions are usually related to low *Leishmania* parasite burden and impaired parasite clearance. In BALB/c mice tissue damage due to CL lesions is extensive and *Leishmania* replication is unrestricted. Thus, high burden of parasites trigger Th2-biased immune responses and production of distinct cytokines such as IL-10 which results in necrosis and fibrosis due to chronic tissue remodeling [39, 40]. Therefore, it is suggested to evaluate the effects of NAI therapy on human CL lesions.

In conclusion, this study demonstrates that NAIs exposure directly affect *L*. *major* promastigotes and intracellular amastigotes *in vitro*, and *in vivo* studies showed that NAIs therapy cures CL lesions and reduces the parasite burden in *L*. *major* infected mice. NAIs therapy also impacts on the immune response, by inducing a TH1-biased cellular response. These results underline the potential of NAI therapy as a promising alternative treatment for CL caused by *L*. *major*. NAIs treatment is non-invasive, inexpensive, safe and easy to use. However, more critical studies need to be carried out on the direct and indirect effects on NAIs on humans and other animal models, the mechanisms by which NAIs therapy increase cellular immune responses, and how NAIs directly act on these parasites. Ongoing research will provide insights into these aspects.
